# Anisotropic fracture mechanics of pre-cracked T_4,4,4_-Graphyne Nanosheets: Effects of crack geometry and temperature

**DOI:** 10.1371/journal.pone.0329337

**Published:** 2026-03-10

**Authors:** Chuanyuan Tan, Ali Ghasemi

**Affiliations:** 1 Han’s Laser Corp., Katy, Texas, United States of America; 2 Advanced Research and Development Center, LIPS Research Foundation, European International University, Paris, France; IGDTUW: Indira Gandhi Delhi Technical University for Women, INDIA

## Abstract

An atomistic study is conducted to elucidate the fracture behavior of pristine and centrally pre-cracked T_4,4,4_-graphyne nanosheets (150 Å × 150 Å) under uniaxial tension in both X- and Y-directions. Stress–strain responses are analyzed as functions of crack length (30–60 Å), orientation (0°–90°), and temperature (200–1000 K). Elastic modulus degradation is captured by power-law and trigonometric models, yielding high correlation coefficients. Ultimate tensile strength and fracture strain are shown to decline with increasing crack length and temperature, while toughness and mode I fracture toughness illustrate anisotropic energy absorption and crack-tip shielding effects, particularly under X-loading where ligament bridging and bond rotation mechanisms are activated. Thermal softening is modeled via the Wachtman equation, revealing near-linear modulus reduction and an inversion of directional stiffness at elevated temperatures. The results demonstrate that crack-length thresholds (~30% of sheet width) and mixed-mode loading conditions critically govern the transition from ductile-like to brittle fracture regimes in anisotropic 2D graphyne nanosheets.

## 1. Introduction

Graphene’s exceptional mechanical strength, thermal conductivity, and charge carrier mobility have positioned it as a transformative material. However, its semimetallic nature with a zero bandgap severely limits its utility in digital electronics and optoelectronics, where a switchable “off” state is essential [1–2]. To address this, several bandgap engineering strategies have been developed: (1) Chemical functionalization, such as fluorination or oxidation, disrupts π-conjugation by introducing sp³ bonds, creating insulating graphene oxide (GO; bandgap ∼4.7 eV) or semiconducting reduced GO (rGO; 1.1–1.9 eV) [3]; (2) Nanostructuring via quantum confinement in nanoribbons (<10 nm width) induces bandgaps up to 0.5 eV [4]; and (3) Heterostructures with materials like brucite (Mg(OH)₂) leverage van der Waals interactions to tune electronic properties [5]. Despite these advances, such modifications often compromise graphene’s native properties, prompting exploration of intrinsic semiconducting carbon allotropes like graphynes.

By inserting acetylenic (–C ≡ C–) linkages between hexagons, one obtains the graphyne family of 2D carbon networks [6–7]. Different graphynes are classified by the number of linking units: for example, graphyne (GY) has one triple-bond spacer, graphdiyne (GDY) has two, graphtriyne has three, etc [8]. In these allotropes carbon atoms are mixed sp and sp² hybridized, giving rise to a planar lattice with intrinsic pores. Importantly, many graphynes have nonzero bandgaps. For instance, γ-graphyne and γ-graphdiyne are predicted semiconductors: density-functional calculations find direct gaps of ~0.46–0.48 eV (PBE) which rise to ~0.9–1.0 eV under HSE functionals [9]. In fact, graphyne and graphdiyne networks generally behave as semiconductors (band gaps ~0.5–0.6 eV) [5,9]. By contrast, other graphyne topologies (α, β, δ) can be semimetallic with Dirac points, and still others (e.g., “R-graphyne”) are metallic [5,9]. The structural porosity of graphynes leads to unique applications. Unlike graphene’s uniform lattice, γ-graphyne and its derivatives have regular triangular or hexagonal pores of controllable size. This nano-porosity (with pore diameters on the order of 3–7 Å can selectively filter molecules and ions [5,9]. For example, pristine γ-graphyne-3 (pore ≈6.9 Å) was proposed for seawater desalination, allowing water but blocking hydrated ions [9]. In general, γ-graphyne-n (n ≥ 2) membranes show high water flux and tunable salt rejection, and chemical modification (e.g., hydrogenation) further improves selectivity [9]. Aside from membranes, graphyne derivatives have been explored for energy and sensing. Recent reviews note that graphdiyne shows outstanding performance in catalysis, energy storage (Li/Na batteries, supercapacitors), environmental remediation, and sensing (biosensors, etc.) [10]. Its extended π-network and moderate gap favor carrier mobility, while the acetylenic linkers enhance chemical reactivity. In short, 2D graphynes combine graphene-like conductivity with a built-in bandgap and intrinsic porosity, making them promising for next-generation electronics, membranes and energy devices [9,10].

Molecular dynamics studies have extensively investigated the mechanical properties and fracture mechanisms of various graphyne allotropes. Zhang et al. [11] demonstrated that acetylenic linkages in α-, β-, γ-, and 6,6,12-graphynes significantly reduce fracture stress and Young’s modulus compared to graphene, with mechanical deterioration proportional to the percentage of these linkages. The weak single bonds in acetylenic linkages serve as fracture initiation sites due to low atom density [11]. Temperature and strain-rate sensitivity studies revealed that graphynes are more temperature-sensitive than graphene, with sensitivity correlating to acetylenic linkage percentage [12]. Faria et al. [13] extended this work to γ-graphyne and γ-graphdiyne nanotubes, employing both AIREBO and ReaxFF potentials to analyze tensile behavior and propose fracture mechanisms. Notably, Brommer and Buehler [14] identified a unique delocalized crack propagation mechanism in graphdiyne, where cracks propagate diagonally rather than along their original direction, distinguishing it from graphene’s failure behavior.

A promising addition to the graphyne family, T_4,4,4_-graphyne is a 2D carbon allotrope composed of alternating hexagonal and rectangular rings linked by acetylenic chains [9]. Recent density functional theory (DFT) studies confirm its dynamic and thermal stability, with cohesive energy (~–8.44 eV/atom) lower than β-graphdiyne, indicating greater stability and potential for synthesis [9]. Unlike graphene’s gapless nature, T_4,4,4_-graphyne exhibits a direct bandgap (~0.6–0.7 eV at the M point) arising from pz orbital interactions, as shown by both DFT and tight-binding models [9]. Its uniform nanopores (~6.41 Å) make it especially suited for applications like filtration, similar to γ-graphyne-3 [9]. Compared to other 2D carbons, it offers a balance between moderate electronic bandgap and intrinsic porosity, while its sp-hybridized segments reduce stiffness relative to graphene [9]. Several recent studies have explored the promising properties and applications of T_4,4,4_-graphyne using first-principles and DFT methods. Majidi and Ayesh [15] demonstrated that Na atoms exhibit exceptional mobility on T_4,4,4_-graphyne with a low energy barrier, resulting in a high theoretical Na storage capacity (Na_8_C_9_) that surpasses graphite and other 2D carbon materials, making it a strong candidate for Na-ion battery anodes. Wu et al. [16] investigated Li and Na co-decoration on T_4,4,4_-graphyne, revealing favorable adsorption energies and a high hydrogen uptake capacity (~10.46 wt%), supported by charge density analysis and molecular dynamics (MD) simulations, suggesting its potential for hydrogen storage applications. Further, Majidi [17] studied the adsorption of the mercaptopurine drug on T_4,4,4_-graphyne, finding weak physical adsorption accompanied by modulations in the bandgap sensitive to drug concentration and external electric fields, indicating suitability for drug sensing. Additionally, Majidi and Sarkar [18] examined the adsorption of NO_x_ and CO_x_ gases, showing that while CO and CO_2_ weakly physical absorb without significant electronic changes, NO and NO_2_ adsorption notably alters the electronic properties, highlighting T_4,4,4_-graphyne’s potential as a sensitive gas sensor for nitrogen oxides. Collectively, these studies underscore the multifunctional capabilities of T_4,4,4_-graphyne in energy storage, sensing, and hydrogen storage technologies.

To predict tensile strength and toughness of such 2D carbons, atomistic simulations are essential. DFT provides a rigorous baseline for ideal strength and elastic constants in pristine, small-unit-cell systems at 0 K but is impractical for large-area simulations [19–20]. Although ab initio MD extends DFT to finite temperatures, its high computational cost limits domain size and time scales [21]. Machine-learned interatomic potentials (MLIPs)—such as neural network or tensor-based models—can approach quantum-level accuracy while enabling simulations of larger systems, and have recently demonstrated reliable prediction of fracture toughness and strength in 2D materials, including non-equilibrium and defected regimes [22]. However, MLIPs require extensive, careful training across diverse configurations (strain, crack states, bond breaking). In contrast, classical MD with semi-empirical potentials (e.g., Tersoff, REBO, AIREBO) offers a time-tested compromise: these potentials reliably model bond stretching, angle bending, and breakage in carbon networks, capturing qualitative fracture behaviors—such as crack-tip shielding, ligament bridging, and anisotropic toughness—even though they may underestimate absolute values [23–24]. Moreover, MD enables systematic exploration of parameter space—temperature, crack size, orientation, and strain rate—for systems of tens to hundreds of nanometers, which would be infeasible via DFT or MLIP without substantial new model development. As a result, semi-empirical potentials remain the pragmatic standard for simulating tensile strength and toughness in novel 2D carbon allotropes like T₄,₄,₄-graphyne, offering a reliable platform for predicting failure mechanisms while ensuring computational efficiency.

While T_4,4,4_-graphyne is first theorized by Wang et al. [9] and its electronic/adsorption properties are extensively investigated, its fracture behavior under defect-mediated failure scenarios remains unexplored. This study provides a pioneering atomistic investigation into the anisotropic fracture mechanics of pre-cracked T_4,4,4_-graphyne nanosheets, a domain previously unexplored in the literature. While previous works have characterized the electronic and adsorption properties of T_4,4,4_ graphyne, its mechanical response under defect-mediated failure scenarios, particularly concerning crack geometry and temperature effects, remained largely unknown. Our findings offer a crucial complement to existing knowledge by revealing the unique fracture mechanisms, such as ligament bridging and bond rotation, that govern its mechanical integrity. In comparison to graphene, which exhibits high strength but often brittle fracture, T_4,4,4_-graphyne demonstrates a more complex anisotropic behavior, with a notable brittle-to-quasi-ductile transition influenced by temperature and loading direction. The choice of the AIREBO-M potential, while widely accepted for carbon systems, inherently introduces a degree of uncertainty in absolute quantitative values. However, the observed trends and qualitative mechanisms, such as the anisotropic response to crack orientation and thermal softening, are robust and consistent with fundamental principles of materials science. The systematic exploration of parameter space—including crack length, orientation, and temperature—provides a comprehensive understanding of T_4,4,4_-graphyne’s fracture behavior, distinguishing this work from studies focusing solely on pristine structures or limited defect configurations. This detailed characterization of fracture toughness, ultimate strength, and fracture strain, coupled with insights into crack propagation pathways, establishes a foundational understanding for the design and application of T_4,4,4_-graphyne in next-generation nanodevices where mechanical reliability is paramount.

## 2. T_4,4,4_-graphyne model and computational detail

**Fig 1:**
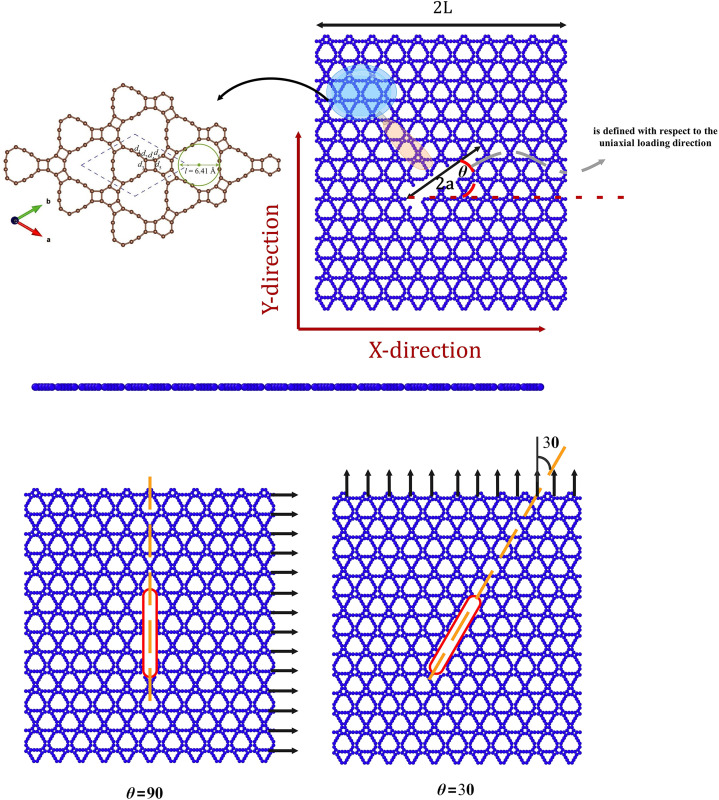
Structural representation of a pre-cracked T_4,4,4_-graphyne nanosheet.

Fig 1 depicts a square pre-cracked T_4,4,4_-graphyne monolayer with a central line crack. T_4,4,4_-graphyne—a 2D trigonal lattice material (plane group *p-6m2*, space group 187)—features hexagonal carbon rings each surrounded by three rectangular tetragonal rings, denoted by the nomenclature “4,4,4”. Its unit cell (lattice constant $\begin{array}{c} a=b=\text{9}.\text{2}\text{9}\text{2}\text{ \textbackslash{}AA} \end{array}$) contains 18 carbon atoms, exhibiting a nanoporous structure. Carbon atoms within triple bonds are sp-hybridized; others are predominantly sp²-hybridized. Bond parameters deviate slightly from ideal sp/sp² hybridization: triple bond length d_1_ = 1.253 Å is notably shorter than d_2_–d_6_ (1.343–1.507 Å), attributed to triple-bond stabilization. Distortions include a single-triple bond angle of 170.3° and non-90° angles in rectangular rings. Bonds d_2_–d₆ remain near graphene’s sp² bond length (1.42 Å). The porous structure exhibits a periodic array of holes of diameter 6.41 Å. a nanoporous structure and the pore diameter of the circumcircle is about 6.41 Å, close to that of bare γ-graphyne-3 (6.9 Å) which has been proposed for seawater desalination [9].

**Fig 2:**
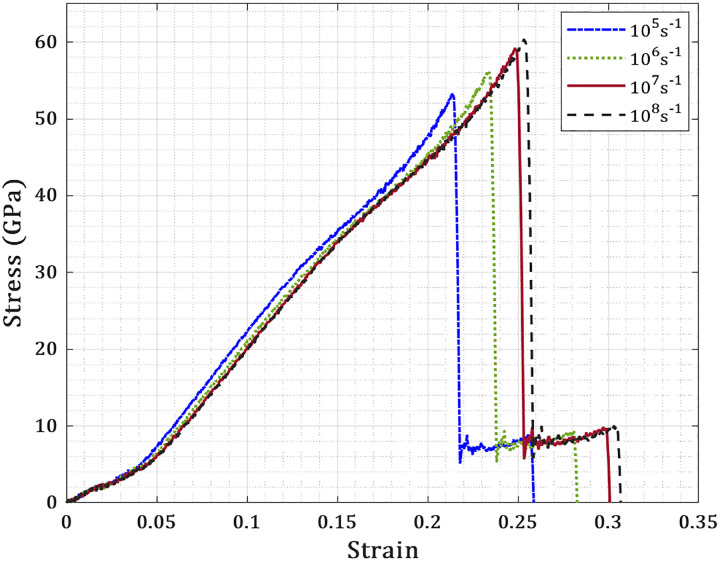
Stress–strain response of a pristine T_4,4,4_-graphyne nanosheet (150 Å  × 150 Å) under uniaxial tension along the X-direction at different strain rates.

The fracture behavior and mechanical response of T_4,4,4_-graphyne nanosheets are analyzed via classical molecular dynamics simulations using the LAMMPS package [25], employing the AIREBO-M potential [26] due to its proven ability to capture bond dissociation and reformation in sp/sp² carbon systems. This force field combines Morse and Lennard-Jones terms, enabling accurate modeling of crack-tip phenomena and anisotropic fracture pathways. Prior to deformation, the nanosheet is equilibrated at 300 K and ambient pressure using an NPT ensemble for 100 ps to eliminate residual stress. Uniaxial tension is then imposed along either principal axis under an NVT ensemble with a Nosé–Hoover thermostat and a constant strain rate of 10⁷ s ⁻ ¹, which ensures convergence of mechanical response. The influence of strain rate on the fracture behavior of T_4,4,4_-graphyne is also examined. Fig 2 presents the stress–strain response of a pristine nanosheet (150 Å × 150 Å) under uniaxial tension along the X-direction at different strain rates. The results show that for strain rates above 10⁷ s ⁻ ¹, the variation in fracture stress and strain is less than 2%, indicating that the overall trends in mechanical response are robust. A time resolution of 0.5 fs permits detailed tracking of atomic rearrangements during fracture. Single-row edge fixation (hinged boundary condition) is used to avoid over-constraining the structure, as multi-row constraints were found to introduce artificial stiffness [27]. The simulation domain (150 Å) is sufficiently large to prevent artificial interactions from periodic boundaries, ensuring that crack-tip fields remain localized. The directional fracture trends and stiffness degradation with crack length follow Griffith scaling and confirm the validity of the model framework [28].

To ensure statistical reliability of the reported mechanical properties, critical simulation conditions are replicated with three independent runs using different random velocity initializations while maintaining identical structural and loading parameters. All reported values represent mean ± standard deviation unless otherwise specified. The uncertainty in derived quantities (e.g., percentage changes, toughness ratios) is calculated using standard error propagation methods. Statistical significance of directional differences was assessed using two-tailed t-tests with α = 0.05.

The mechanical behavior of T_4,4,4_-graphyne is evaluated via molecular dynamics using standard analysis of stress–strain data. The elastic modulus is extracted from the linear region (up to ~3% strain) as the slope of stress versus strain, where engineering stress is calculated using the initial cross-sectional area with a nominal sheet thickness of 3.4 Å. Fracture strain is defined at peak stress and computed as the relative elongation at failure. Toughness, representing energy absorption before fracture, is determined by numerically integrating the stress–strain curve using the trapezoidal rule. Additionally, von Mises stress is used to assess local stress concentrations and potential failure zones. For 2D systems, the standard 3D von Mises equation is reduced by neglecting out-of-plane components, yielding a simplified form involving only in-plane stresses $\begin{array}{c} \left(\sigma_{xx},\sigma_{yy},\sigma_{xy}\;\right) \end{array}$. This 2D formulation captures shear-driven stress localization and provides insight into deformation and fracture patterns during tensile loading.

Uniaxial tensile deformation is applied independently along the two principal in-plane crystallographic axes of the T_4,4,4_-graphyne lattice, referred to as the X and Y directions. The crack orientation angle (θ) is defined relative to the loading direction, with θ = 0° indicating a crack aligned parallel to the applied tensile force, and θ = 90° denoting a crack perpendicular to it. To investigate the influence of crack orientation on the fracture response, additional intermediate angles (e.g., 30°, 45°, and 60°) are also examined, allowing for the assessment of mixed-mode (Mode I/II) fracture behavior. The fracture toughness $\left({\text{K}}_{\text{c}}\right)$ is evaluated as a function of crack orientation, accounting for both pure mode I (opening mode) and mixed-mode (modes I/II) fracture conditions. For the perpendicular case (θ = 90°), crack propagation followed classical mode I behavior, with ${\text{K}}_{\text{c}}$ reducing to the mode I critical stress intensity factor ${\text{K}}_{\text{Ic}}$. At oblique angles, the effective fracture toughness incorporated contributions from both normal and shear stresses. The critical stress intensity factor at failure is calculated at peak tensile stress using a modified finite-width solution [29]:


$${\text{K}}_{\text{c}}=\sigma_{f}\sqrt{\text{2}L\times tan(\frac{\pi a}{\text{2}L}})$$ (1)

where $\sigma_{f}$ is the maximum tensile stress, and 2L is the width of the nanosheet, and 2a denote the initial crack length.

Griffith’s criterion, derived within the framework of linear elastic fracture mechanics (LEFM), was originally formulated for continuum materials. Sohail and Roy [30] demonstrated significant deviations from LEFM-based predictions in amorphous materials below a critical threshold crack length, indicating limited applicability at the nanoscale. Similarly, Lakshmipathy et al. [31] showed that discrete atomic lattice effects lead to deviations from LEFM displacement fields, attributing these to geometrical nonlinearities rather than material nonlinearity. However, studies on crystalline materials show more promising results. Jin and Yuan [32] found good agreement between atomistic simulations and continuum mechanics for energy release rates in graphene systems, with atomic stress distributions matching linear elastic solutions well. Tsai and Sie [33] demonstrated that stress intensity factors calculated using Hardy stress formulation showed compatibility with finite element analysis, particularly for larger crack sizes. As highlighted in [34], rigorous quantitative evaluation of fracture toughness requires convergence toward macroscopic dimensions, and the avoidance of centrally located cracks that can violate the assumptions underlying Stroh’s formalism. Our approach, designed for qualitative comparative purposes, does not satisfy these stringent criteria and thus does not yield absolute $K_{Ic}$ values. Nevertheless, it provides atomistically informed insights into trends in fracture behavior, particularly regarding the influence of crack geometry and temperature on the mechanical response of T_4,4,4_-Graphyne Nanosheets.

## 3. Findings and discussion

### 3.1. Fracture process

**Fig 3:**
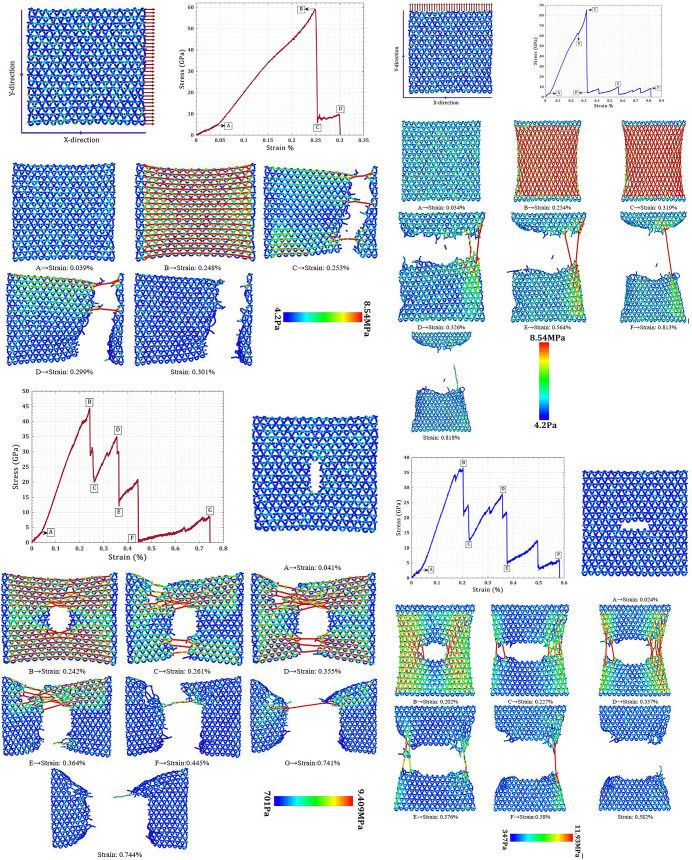
Fracture evolution of T_4,4,4_-graphyne nanosheets (150 Å  × 150 Å) under uniaxial tension: (a) pristine X-loading, (b) pristine Y-loading, (c) pre-cracked X-loading, and (d) pre-cracked Y-loading. Pre-cracks (40 Å) are introduced perpendicular to the loading axis.

Fig 3 presents a comparative atomistic visualization of the fracture process in T_4,4,4_-graphyne nanosheets subjected to uniaxial tensile loading in both pristine and centrally pre-cracked configurations along the X- and Y-directions. Each nanosheet is modeled as a square membrane with a lateral dimension of 150 Å × 150 Å, consisting of a periodic 2D network of carbon atoms with sp and sp² hybridized bonding. In the pre-cracked cases, a linear crack of 40 Å length is introduced at the center of the nanosheet, aligned perpendicular to the direction of applied loading—ensuring a pure mode-I tensile fracture condition.

In Fig 3(a), In the pristine state, the fracture behavior of T_4,4,4_-graphyne exhibits strong directional dependence. When loaded along the X-direction, failure initiates and propagates along the Y-direction, specifically near the loaded edge (where displacement is applied), indicating stress concentration near boundary constraints. In contrast, under loading along the Y-direction, fracture initiates along the X-direction, slightly offset from the edge and closer to the midline, suggesting a more distributed stress field and delayed crack nucleation (Fig 3(b)). From a mechanical standpoint, this anisotropic fracture response reflects the directionally dependent atomic connectivity and bonding topology inherent in the T_4,4,4_-graphyne lattice. The higher ultimate stress observed under Y-direction loading (85.4 ± 4.1 GPa) compared to X-direction (59.2 ± 2.8 GPa) suggests that the bond arrangement and density along the Y-direction offers greater load transfer capability and resistance to bond rupture. Furthermore, the strain at peak stress is 0.253 ± 0.018 (X) and 0.326 ± 0.024 (Y), with final fracture strains extending to 0.301 ± 0.021 and 0.818 ± 0.058, respectively. The substantially greater fracture strain in the Y-direction indicates a more ductile-like or crack-arresting behavior, likely facilitated by rebonding and bond rotation mechanisms that dissipate energy during deformation.

As shown in Fig 3(c), in the X-loading case of the pre-cracked T_4,4,4_-graphyne nanosheet, the fracture path diverges from the predefined crack line and propagates asymmetrically through the lattice, indicating that the local stress intensity at the crack tip is insufficient to sustain direct cleavage. Instead, crack growth is deflected into the surrounding matrix, leading to the formation of tortuous fracture surfaces accompanied by ligament bridging and crack blunting. This behavior is attributed to the interplay between the anisotropic bond distribution and the structural flexibility provided by acetylenic linkages, which enable localized energy absorption and hinder linear crack propagation. As a result, the material exhibits a significantly extended fracture strain of 0.744 ± 0.052—a 147 ± 8% increase compared to the pristine case—despite a reduction in ultimate tensile strength to 44.5 ± 2.1 GPa. The stress–strain curve reveals marked fluctuations, consistent with discrete bond rupture events and localized plasticity that arise as the structure undergoes non-uniform deformation. These features collectively indicate the activation of crack-shielding mechanisms, including stress redistribution and progressive bond rearrangement, which enhance the material’s resistance to catastrophic failure under tensile loading in the X-direction.

Fig 3(d) illustrates the fracture behavior of the pre-cracked T_4,4,4_-graphyne nanosheet under Y-direction loading, where the pre-existing crack is oriented along the X-direction (horizontal axis). In this configuration, the crack lies perpendicular to the direction of applied uniaxial tensile strain, producing a classical mode-I crack opening condition. Unlike the X-loading case, here the crack propagation follows the initial crack plane with remarkable precision, indicating a dominant role of crack-tip stress concentration in directing failure. This behavior reflects a weaker in-plane fracture resistance in the horizontal atomic arrangement, which lacks sufficient lattice deflection or shielding to arrest or divert crack growth. As the applied strain increases, the crack gradually extends along the X-direction, accompanied by bond rupture that initiates at the crack tip and propagates in a near-linear fashion through the weakened carbon framework. Compared to the pristine Y-loading case, the introduction of the crack leads to a significant reduction in ultimate tensile strength (from 85.4 ± 4.1 GPa to 36.5 ± 2.2 GPa) and a moderate decrease in fracture strain (from 0.818 ± 0.058 to 0.578 ± 0.041), clearly demonstrating the embrittling effect of the defect. Despite this degradation, the stress–strain curve exhibits noticeable fluctuations, which suggest intermittent bond-breaking and rebonding events, especially near the acetylenic chains adjacent to the crack tip. These dynamic bond rearrangements contribute to localized energy dissipation and slightly delay the final catastrophic failure.

### 3.2. Effect of crack length

**Fig 4:**
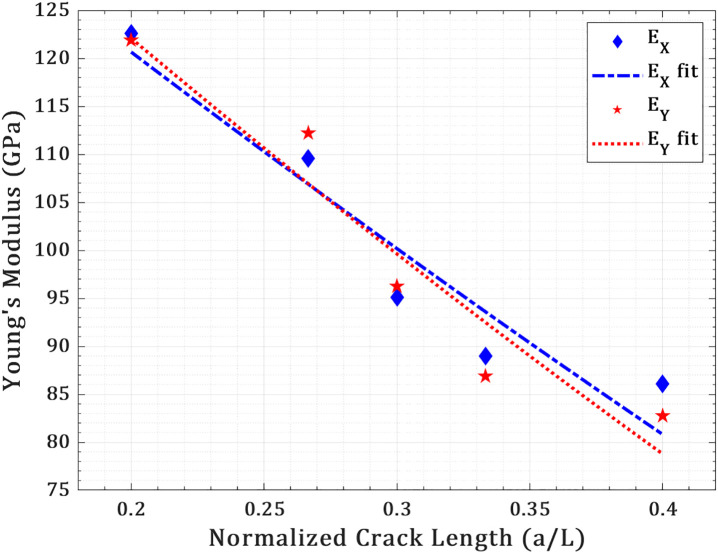
Young’s modulus of pre-cracked T_4,4,4_-graphyne nanosheets under uniaxial loading at 300 K as a function of crack length in the X- and Y-directions.

The influence of central crack length on the in-plane elastic modulus of a square T_4,4,4_-graphyne nanosheet (150 Å × 150 Å) is evaluated under uniaxial tension along both the X- and Y-directions at a constant temperature of 300 K, as shown in Fig 4. A line crack, placed centrally and oriented perpendicular to the loading direction, is incrementally varied in length from 30 Å to 60 Å to assess its impact on the stiffness response. In the pristine state, the elastic modulus is determined to be 118.2 ± 3.1 GPa along the X-direction and 126.2 ± 3.4 GPa along the Y-direction, indicating moderate in-plane anisotropy inherent to the lattice topology of T_4,4,4_-graphyne.

For comparison, the Young’s modulus of single-layer graphene has been reported as approximately 0.8 TPa, calculated within the initial 5% strain range [35]. Similarly, phagraphene nanosheets exhibit an average elastic modulus close to 0.8 TPa [36]. Molecular dynamics simulations for various graphyne structures indicate Young’s modulus values ranging from 532 GPa to 700 GPa, depending on crystallographic direction [37]. Specifically, perfect γ-graphyne demonstrates directional anisotropy, with a Young’s modulus of approximately 0.586 TPa along the reclined chair direction and about 0.510 TPa along the zigzag direction [38]. For α-graphyne and related allotropes, the elastic modulus generally spans 0.12–0.5 TPa, reflecting the influence of structural configuration on mechanical stiffness [39].

As crack length increases, a clear degradation in stiffness is observed in both directions. For the X-direction, the modulus decreases from 104.2 ± 2.8 GPa at 30 Å crack length to 58.9 ± 2.1 GPa at 60 Å. In the Y-direction, a similar but slightly more pronounced decline is recorded—from 114.0 ± 3.2 GPa at 30 Å to 61.7 ± 2.4 GPa at 60 Å. The decline in modulus with increasing crack length is attributed to the progressive removal of load-bearing pathways and redistribution of local stress fields around the crack tip, which weakens the structural connectivity across the tension axis. Interestingly, for all crack lengths, the Y-direction modulus remains consistently higher than the X-direction modulus, reflecting the higher intrinsic stiffness and superior atomic connectivity along that axis in the T_4,4,4_ lattice. Cracks extending beyond 30% of the nanosheet width (≈45 Å) are observed to significantly disrupt the sp–sp² hybridized carbon network, marking the onset of a mechanical percolation threshold. At this scale, stress no longer transfers through a continuous lattice but instead reroutes along weaker, non-bonded van der Waals corridors, resulting in increased structural compliance. This transition is particularly evident in the X-loading configuration, where crack-tip blunting and deflected fracture pathways introduce tortuous microvoids and local porosity, thereby accelerating the reduction in effective stiffness. Conversely, in the Y-loading case, the pre-existing crack promotes a more brittle, linear propagation, concentrating stress along a direct cleavage plane and facilitating abrupt bond rupture with less energy dissipation. This distinction reflects the anisotropic fracture resistance of T_4,4,4_-graphyne, where local bond topology and crack–lattice interactions jointly dictate mechanical degradation.

The generalized power-law model $\text{E}={\text{E}}_{\text{0}}{\left(\text{1}-\frac{\text{a}}{h}\right)}^{n}$ is used to describe the reduction in elastic modulus with increasing normalized crack length in anisotropic materials [40]. In this study, it effectively captures the nonlinear stiffness degradation in the T4,4,4-graphyne nanosheet. The best-fit expressions are ${\text{E}}_{X}=\text{164}.\text{65}{\left(\text{1}-\frac{\text{a}}{L}\right)}^{\text{1}.\text{39}}$ (${\text{R}}^{\text{2}}=\text{0}.\text{91}$) and ${\text{E}}_{Y}=\text{171}.\text{49}{\left(\text{1}-\frac{\text{a}}{L}\right)}^{\text{1}.\text{52}},$ (${\text{R}}^{\text{2}}=\text{0}.\text{92}$), indicating strong agreement with simulation data. The slightly higher exponent in the Y-direction reflects a more pronounced stiffness loss per unit crack growth, consistent with directional mechanical anisotropy.

**Fig 5:**
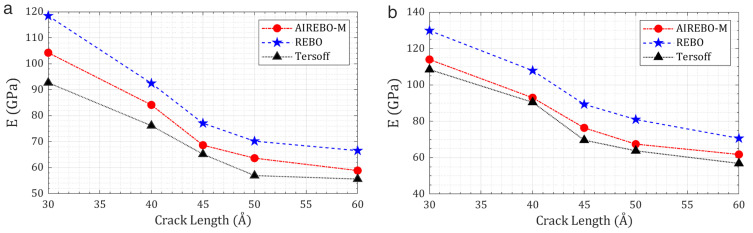
Comparative variation of elastic modulus with crack length in T_4,4,4_-graphyne nanosheets under uniaxial loading: (a) X-direction and (b) Y-direction, predicted using AIREBO-M, REBO, and Tersoff potentials.

To verify the robustness of the findings, cross-validation is conducted using REBO and Tersoff potentials for representative cases. Although the absolute magnitudes of the elastic modulus are found to differ among the potentials, the overall anisotropic trends with respect to crack length and loading direction are consistently preserved. This agreement across parameterizations confirms that the reported anisotropic fracture responses of T_4,4,4_-graphyne nanosheets are not artifacts of a specific potential choice, but instead are intrinsic material characteristics. Fig 5 presents a comparative summary of the elastic modulus versus crack length under X- and Y-loading directions as obtained from AIREBO-M, REBO, and Tersoff, demonstrating that anisotropic behavior persists independent of the potential employed.

**Fig 6:**
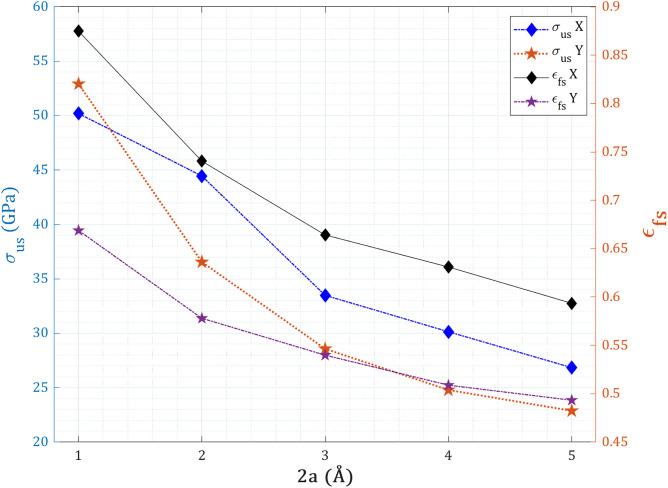
Ultimate tensile strength and fracture strain of pre-cracked T_4,4,4_-graphyne nanosheets with increasing central crack length (perpendicular to loading) under uniaxial tension in the X- and Y-directions.

Fig 6 presents the evolution of ultimate tensile strength and fracture strain of centrally pre-cracked T_4,4,4_-graphyne nanosheets under uniaxial tension in both X- and Y-directions as a function of crack length ranging from 30 Å to 60 Å. The results clearly demonstrate that the introduction and growth of a central crack significantly weaken the mechanical performance in both directions. Under X-direction loading, where the central crack is oriented perpendicular to the applied strain, the ultimate tensile strength decreases markedly from 51.2 ± 2.4 GPa (at 30 Å crack length) to 26.8 ± 1.6 GPa (at 60 Å), a significant reduction compared to the pristine strength of 59.2 ± 2.8 GPa. This decline is governed by crack-tip blunting and the formation of ligament bridges, which facilitate stress redistribution through localized acetylenic chain rotation and bond stretching. These mechanisms introduce nanoscale porosity, reducing effective load transfer while simultaneously enhancing fracture strain. Notably, the fracture strain increases from 0.301 ± 0.021 in the pristine state to 0.875 ± 0.061 at 30 Å crack length—a remarkable 191 ± 12% enhancement—due to tortuous crack propagation, sequential bond rupture, and localized plasticity that delay catastrophic failure. In contrast, under Y-direction loading, the system undergoes a more brittle fracture process. As the crack lies perpendicular to the Y-strain axis (parallel to acetylenic chains), failure occurs via linearly propagating cracks with minimal deflection. This promotes stress concentration and direct sp–sp² bond cleavage, leading to a drop in ultimate stress from 52.9 ± 2.7 GPa to 22.9 ± 1.4 GPa, and a concurrent decrease in fracture strain from 0.669 ± 0.048 to 0.493 ± 0.035, both of which fall below the pristine Y-direction values (85 GPa, 0.818, respectively). Importantly, when the crack length exceeds 45 Å (~30% of sheet width), the distinction between X and Y fracture responses diminishes significantly: at 60 Å, the ultimate stress in the X-direction (27 GPa) and Y-direction (23 GPa) differ by only 4 GPa, indicating a substantial loss of anisotropic fracture resistance. This suggests that beyond a critical defect size, the elasticity of the acetylenic framework is overwhelmed, and stress pathways transition to weaker van der Waals interfaces, triggering a percolative failure regime. In this regime, both strength and ductility degrade concurrently, approaching isotropic mechanical limits and highlighting the threshold at which structural integrity is universally compromised.

**Fig 7:**
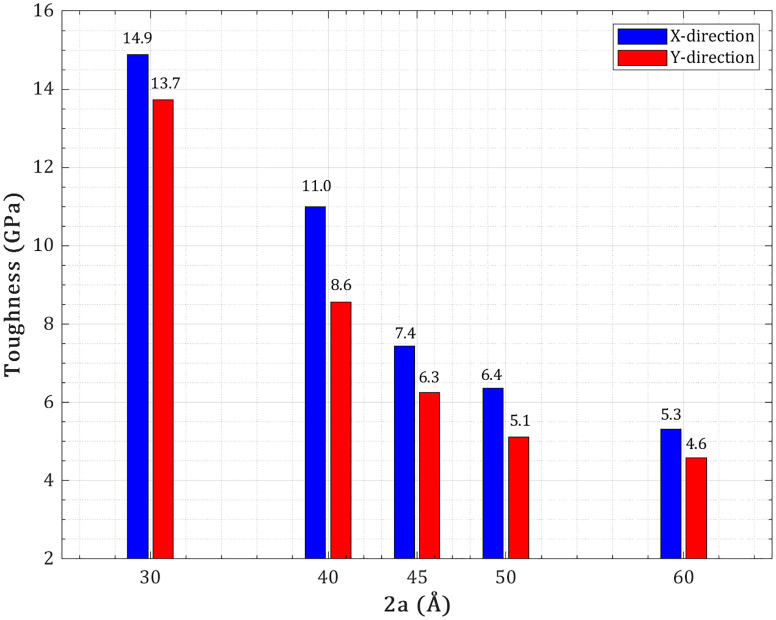
Toughness of pre-cracked T_4,4,4_-graphyne nanosheets with increasing central crack length under uniaxial tension in the X- and Y-directions.

Fig 7 illustrates the variation in toughness, defined as the area under the stress–strain curve and representing the energy absorption capacity prior to fracture, for pre-cracked T_4,4,4_-graphyne nanosheets subjected to uniaxial loading along the X- and Y-directions at 300 K. In the pristine state, the nanosheet exhibits a toughness of 7.17 ± 0.41 GPa and 15.43 ± 0.89 GPa in the X and Y directions, respectively, reflecting an intrinsically higher ductility and energy dissipation ability under Y-direction loading. For X-direction loading (with the crack perpendicular to the applied strain), the toughness initially increases sharply to 14.89 ± 0.76 GPa at a 30 Å crack length, representing a 108 ± 7% enhancement over the pristine value. This increase is attributed to tortuous crack deflection, ligament bridging, and progressive bond rupture mechanisms, which enable the system to dissipate mechanical energy efficiently through acetylenic chain rotation, crack-tip blunting, and localized plastic deformation. These mechanisms collectively delay catastrophic failure by distributing stress across multiple deformation sites. However, as crack length exceeds 30 Å, the toughness in the X-direction decreases steeply—falling to 5.3 GPa at 60 Å, which is a 26% reduction below the pristine state. This behavior suggests that longer cracks surpass a critical deflection length, leading to more homogeneous strain fields, reduced stress shielding, and limited ability to sustain distributed damage. In contrast, Y-direction loading shows a monotonic decline in toughness from 13.7 GPa (30 Å) to 4.6 GPa (60 Å), representing a 70% loss relative to the pristine toughness of 15.43 GPa. This trend indicates that linear crack propagation dominates in the Y-direction, with minimal deflection or crack-bridging mechanisms. Here, the acetylenic chains aligned with the loading axis act as brittle “fuse-links”, breaking abruptly under localized tensile stress without triggering plasticity or alternative energy-dissipation modes. Importantly, when the crack length exceeds 45 Å (i.e., 30% of the sheet width), the toughness values in both directions converge to similarly low levels—5.3 GPa in X and 4.6 GPa in Y—indicating a near-complete loss of anisotropic fracture resistance. At this scale, the lattice undergoes a transition to a percolation-controlled failure regime, in which severed load paths force stress redistribution through weaker secondary (van der Waals or strained sp–sp²) interactions. As a result, the energy absorption capacity collapses, even though some residual ductility remains in the X-direction due to non-linear crack propagation.

**Fig 8:**
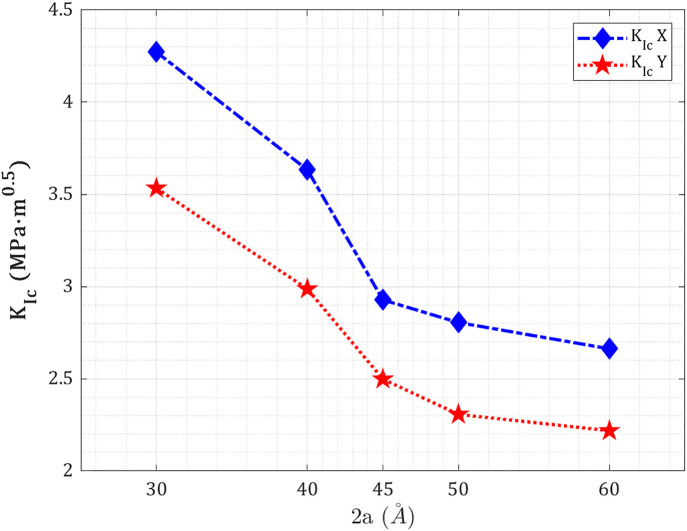
Mode I fracture toughness of pre-cracked T_4,4,4_-graphyne nanosheets with increasing central crack length under Mode I uniaxial loading.

Fig 8 presents the variation of mode-I fracture toughness (${\text{K}}_{\text{c}}\equiv {\text{K}}_{\text{Ic}})$—a critical material parameter describing resistance to crack propagation under pure Mode I loading—with increasing central crack length in T_4,4,4_-graphyne nanosheets subjected to uniaxial tension along the X- and Y-directions at 300 K. The mode I fracture toughness is computed from the peak stress and crack geometry using the classical linear elastic fracture mechanics relation adapted for 2D materials. In this study, cracks are placed perpendicular to the loading axis, satisfying pure Mode I (opening mode) conditions at $\theta ={\text{90}}^{\circ }$, such that the stress intensity factor K directly reflects the magnitude of the singular stress field near the crack tip, and mode I fracture toughness ${\text{K}}_{\text{Ic}}$ denotes the critical threshold for crack initiation.

Pre-cracked T_4,4,4_-graphyne exhibits a sustained anisotropy reversal in mode I fracture toughness relative to its pristine mechanical strength. In the defect-free configuration, the Y-direction exhibits higher tensile strength and toughness. However, with the introduction of a central crack, mode I fracture toughness in the X-direction consistently exceeds that in the Y-direction across all crack lengths studied (30–60 Å). At 30 Å, this anisotropy is most pronounced: marking a significant deviation from the pristine hierarchy. This is attributed to crack-tip shielding mechanisms active under X-loading: acetylenic chains-oriented transverse to the loading axis accommodate ligament bridging, rotational bond plasticity, and tortuous crack deflection, which collectively blunt the stress singularity and raise the energy threshold for crack propagation. In contrast, the Y-direction shows a more brittle response due to linearly propagating cracks aligned with the acetylenic chains, which localize stress and promote rapid failure. As crack length increases, values in both directions decrease significantly.

In comparison with other nanomaterials, monolayer graphene exhibits pronounced anisotropy in its fracture toughness, with values of approximately 3.63 $\text{MPa}\cdot \sqrt{\text{m}}$ along the zigzag direction and 2.96 $\text{MPa}\cdot \sqrt{\text{m}}$ along the armchair direction, as determined via molecular dynamics simulations employing the AIREBO potential [41]. Experimental measurements for graphene fracture toughness typically range from 4 to 5 $\text{MPa}\cdot \sqrt{\text{m}}$ [42]. Bi- and polycrystalline graphene display a wider range of fracture toughness, from 2.5 to 4.5 $\text{MPa}\cdot \sqrt{\text{m}}$, depending on the specific grain boundary structures and misorientation angles [43]. In contrast, single-walled carbon nanotubes exhibit a comparatively lower and more isotropic fracture toughness of approximately 2.7 $\text{MPa}\cdot \sqrt{\text{m}}$, underscoring the critical role of dimensional confinement and structural morphology in governing nanomechanical behavior [40]. γ-Graphyne shows fracture toughness ${\text{K}}_{\text{Ic}}$ values around 2.0 to 2.9 $\text{MPa}\cdot \sqrt{\text{m}}$ from recent nanofracture studies, which are relatively higher among graphyne structures [44].

### 3.3. Effect of crack orientation

**Fig 9:**
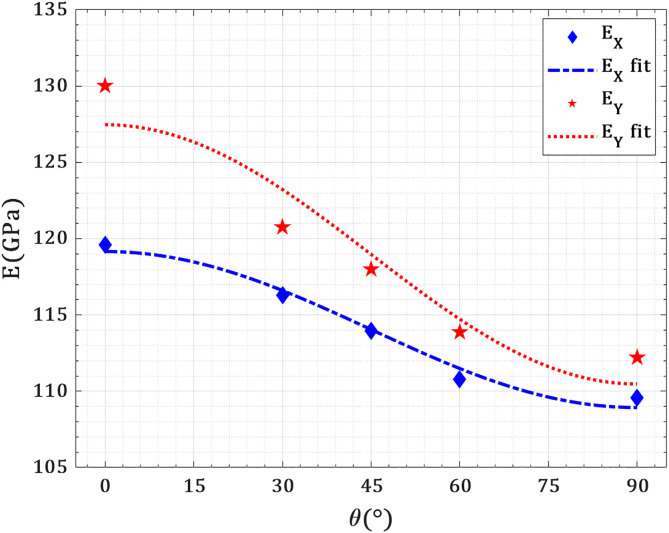
Elastic modulus of pre-cracked T_4,4,4_-graphyne nanosheets as a function of crack orientation angle.

Fig 9 presents the variation of the elastic modulus in both X- and Y-loading directions as a function of crack orientation angle (θ = 0° to 90°) for a fixed crack length of 40 Å. The nanosheet under consideration is a 150 Å × 150 Å pre-cracked T_4,4,4_-graphyne sheet subjected to uniaxial tension, with the crack initially aligned parallel to the loading axis (θ = 0°) and incrementally rotated toward perpendicular alignment (θ = 90°). As the crack orientation angle increases, the elastic modulus in both directions decreases, but the rate of reduction is direction-dependent. For X-loading, the modulus drops from 96 GPa (θ = 0°) to 84 GPa (θ = 90°), while in Y-loading, it decreases from 108 GPa to 93 GPa over the same angular range. This behavior reflects a progressive weakening of the load-bearing network as the crack becomes more aligned with the tensile stress direction. Notably, the elastic modulus values converge asymptotically as θ approaches 90°, corresponding to pure Mode I loading, where the crack lies fully perpendicular to the loading axis and primarily opens under tensile stress without significant shear. At lower crack angles, the crack orientation introduces mixed-mode I/II conditions, where both tensile (mode I) and in-plane shear (mode II) components are active. Under such mixed-mode conditions, the material exhibits enhanced stiffness, as the crack-tip fields are less effective at stress localization due to the combined shearing action, leading to higher effective modulus. This mixed-mode loading disperses the stress concentration and delays localized deformation, preserving more of the pristine lattice’s stiffness. As the crack approaches θ = 90°, the Mode II component vanishes, and the system transitions to a pure opening mode. In this regime, the crack becomes an efficient stress concentrator aligned normal to the loading axis, resulting in reduced lattice connectivity across the loading direction and a drop in elastic response.

The angular dependence of elastic modulus in anisotropic or cracked materials can be accurately captured using trigonometric expressions derived from Fourier series expansions, which effectively represent the periodic and symmetric nature of the elastic response relative to crack orientation [45]. In this study, cosine-based formulations are employed to model this behavior. For the X-direction, the elastic modulus is fitted as ${\text{E}}_{\text{X}}=\text{114}.\text{04}+\text{5}.\text{11 cos}(2\theta )$ with high correlation $\left({\text{R}}^{\text{2}}=\text{0}.\text{98}\right),$ while for the Y direction ${\text{E}}_{\text{Y}}=\text{118}.\text{97}+\text{8}.\text{5}\text{ cos}(2\theta \text{provides a similarly strong fit }\left({\text{R}}^{\text{2}}=\text{0}.\text{91}\right)$. These results confirm that the directional stiffness variation due to crack orientation can be effectively modeled using the symmetric properties captured by trigonometric series, consistent with the theory for anisotropic media.

**Fig 10:**
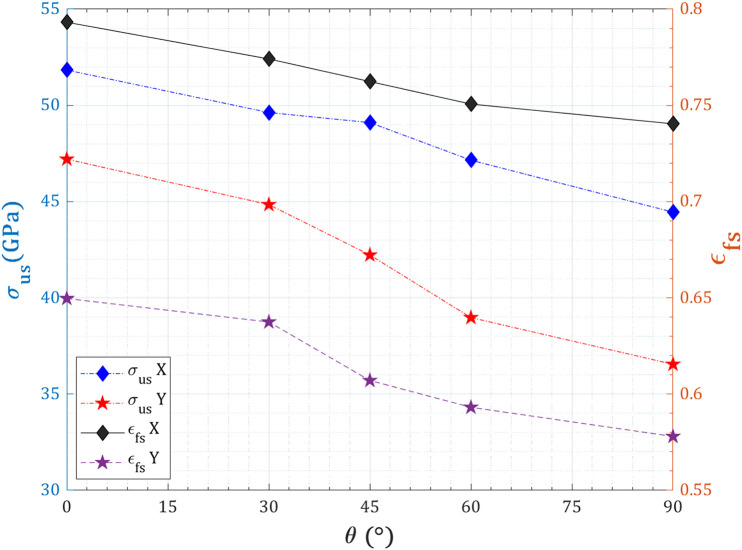
Angular dependence of ultimate strength and fracture strain of pre-cracked T_4,4,4_-graphyne.

The variation of ultimate stress and fracture strain with respect to crack orientation angle (θ = 0°–90°) for a pre-cracked T_4,4,4_-graphyne nanosheet with a fixed central crack length of 40 Å is shown in Fig 10. Under both X- and Y-direction loading, a general declining trend is observed in both ultimate stress and fracture strain as the crack angle increases toward 90°, where the crack becomes fully perpendicular to the loading direction (pure Mode I condition). In the X-loading case, ultimate stress decreases from 51.8 ± 2.6 GPa at θ = 0° to 44.5 ± 2.1 GPa at θ = 90°, representing a 14.1 ± 0.8% reduction. Similarly, fracture strain drops from 0.793 ± 0.055 to 0.740 ± 0.051, indicating a 6.7 ± 0.9% loss in ductility. The higher values at lower crack angles are attributed to mixed-mode loading conditions (Mode I/II), where the shear component mitigates stress concentration at the crack tip, allowing more distributed deformation and delaying catastrophic fracture. In the Y-loading direction, the effects are more pronounced: ultimate stress declines from 47.2 ± 2.4 GPa to 36.5 ± 2.2 GPa (a 22.7 ± 1.2% reduction), while fracture strain falls from 0.650 ± 0.046 to 0.578 ± 0.041 (11.1 ± 1.0% decrease). This sharper decline is due to the alignment of the crack with the acetylenic chains and the absence of toughening mechanisms such as ligament bridging or bond rotation in this configuration. As the crack becomes perpendicular to the tensile axis, stress concentration increases and fracture occurs more abruptly, particularly in the brittle Y-aligned carbon framework.

**Fig 11:**
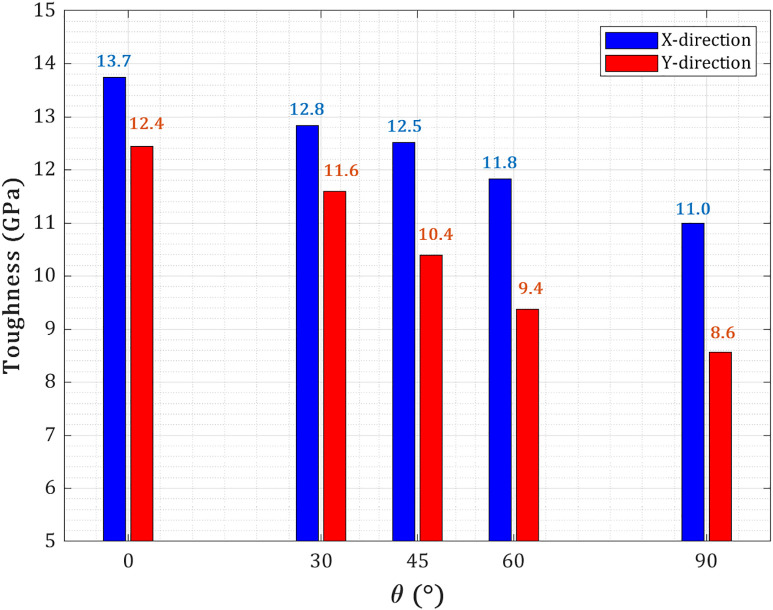
Toughness of pre-cracked T_4,4,4_-graphyne nanosheets as a function of crack orientation angle.

Fig 11 illustrates the variation of toughness—defined as the energy absorption capacity per unit volume before failure—with respect to crack angle (θ = 0° to 90°) for a centrally pre-cracked T_4,4,4_-graphyne nanosheet (150 Å × 150 Å) with a fixed crack length of 40 Å, subjected to uniaxial loading along both the X and Y directions. For both loading orientations, toughness shows a monotonic decrease as the crack angle increases toward 90°, where the crack is fully perpendicular to the tensile axis (pure Mode I). In X-loading, toughness decreases from 13.7 GPa at θ = 0° to 11.8 GPa at 60° and further to 11 GPa at θ = 90°, reflecting a 20% reduction relative to the minimum angle. This decline is attributed to reduced contributions from ligament bridging, acetylenic chain rotation, and crack-tip deflection, which are more effective under mixed-mode (mode I/II) conditions at low crack angles. As the crack becomes orthogonal to the applied strain, these toughening mechanisms are suppressed, and fracture occurs more directly, lowering energy dissipation. A similar but more pronounced trend is observed in Y-loading, where toughness falls from 12.4 GPa at θ = 0° to 8.6 GPa at θ = 90°, signifying a 31% reduction. The steeper decline in Y-direction toughness stems from the more brittle fracture behavior of the sp–sp² bonded carbon network aligned with the loading axis. As crack orientation transitions to mode I, stress localizes more efficiently at the crack tip without significant redistribution, thereby reducing the capacity for plastic-like energy absorption. In both loading cases, the reduction in toughness with increasing crack angle highlights the transition from distributed, ductile fracture behavior at low θ to localized, brittle failure near θ = 90°.

**Fig 12:**
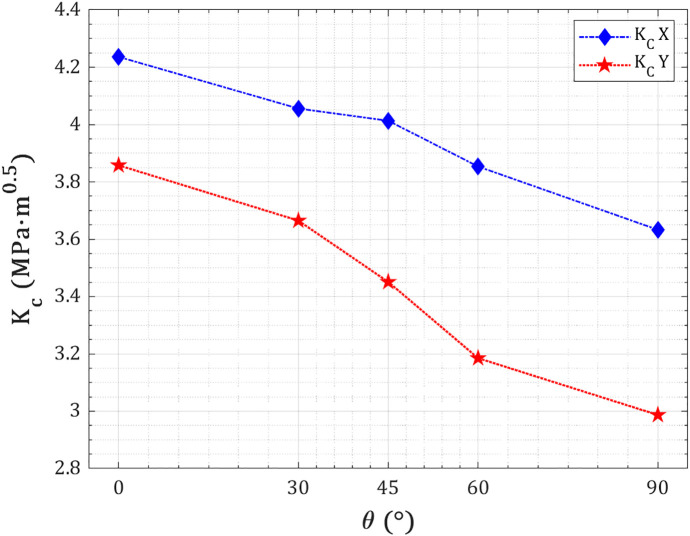
Fracture toughness of pre-cracked T_4,4,4_-graphyne nanosheets with a 40 Å crack as a function of crack orientation angle.

At a fixed crack length of 40 Å and temperature of 300 K, the fracture toughness of the pre-cracked T_4,4,4-_graphyne nanosheet decreases with increasing crack angle, demonstrating the sensitivity of crack resistance to crack orientation relative to the loading axis (as shown in Fig 12). For both X- and Y-direction loading, the highest toughness is observed at 0°—when the crack is parallel to the loading direction—reaching ~4.24 MPa.m^0.5^ in the X-direction and ~3.86 MPa.m^0.5^ in the Y-direction. As the angle increases toward 90°, where the crack is perpendicular to the loading, the fracture toughness drops to ~3.63 MPa.m^0.5^ and ~2.99 MPa.m^0.5^, respectively. This reduction is physically attributed to a transition from a predominantly shear-driven fracture mode (mode II) at lower angles to a more opening-dominated mode I fracture at higher angles, where stress concentration at the crack tip becomes more severe. The sharper decline in the Y-direction implies a weaker shear resistance in that lattice orientation, reinforcing the material’s anisotropic mechanical character governed by its bond configuration and directional load transfer efficiency.

### 3.4. Effect of temperature

**Fig 13:**
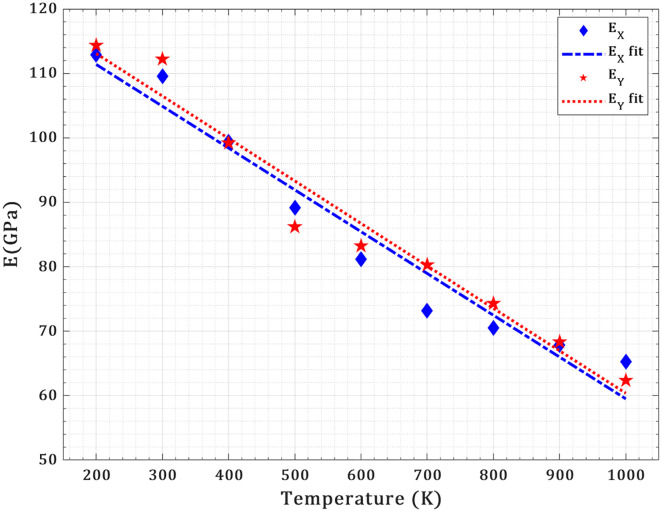
Temperature dependence of the elastic modulus of a pre-cracked T_4,4,4_-graphyne nanosheet. The data are fitted using the Wachtman equation, showing nearly linear thermal softening and directional anisotropy.

The pre-cracked T_4,4,4_-graphyne nanosheet (150 Å × 150 Å with a 40 Å central crack perpendicular to loading) shows a pronounced thermal softening: ${\text{E}}_{\text{X}}$ drops from 86.1 ± 2.4 GPa at 200 K to 42.3 ± 1.8 GPa at 1000 K, while ${\text{E}}_{\text{Y}}$ decreases from 93.0 ± 2.7 GPa to 41.2 ± 1.9 GPa over the same range (as shown in Fig. 13). At low temperatures (200–300 K), ${\text{E}}_{\text{Y}}$ exceeds ${\text{E}}_{\text{X}}$ by ~7–8 GPa, reflecting stronger bond orientations under Y-loading and sharper stress concentration along X. As temperature rises, increased atomic vibrations weaken all bonds, and thermally activated bond‐rearrangement and crack‐tip blunting reduce anisotropy, narrowing the gap by ~500–900 K; above ~900 K, subtle anisotropic thermal expansion and local bond reorganization near the crack even invert the stiffness hierarchy, yielding ${\text{E}}_{\text{X}}$ slightly above ${\text{E}}_{\text{Y}}$ at 1000 K. The thermal softening behavior is further analyzed using the Wachtman equation, a semi-empirical model that captures temperature-dependent reductions in elastic modulus [46]. The fitted expressions—${\text{E}}_{\text{X}}(\text{T})=\text{124}.\text{36}-\text{0}.\text{06T }{\text{e}}^{-\frac{\text{0}.\text{002}}{\text{T}}}\;\left({\text{R}}^{\text{2}}=\text{0}.\text{95}\right)$ and ${\text{E}}_{\text{Y}}(\text{T})=\text{126}.\text{24}-\text{0}.\text{07T }{\text{e}}^{-\frac{\text{0}.\text{003}}{\text{T}}}\;\left({\text{R}}^{\text{2}}=\text{0}.\text{96}\right)$—demonstrate good agreement with simulation data and highlight the intrinsic anisotropy of the nanosheet. Although the model includes an exponential decay term, the fitted curves exhibit a nearly linear decline over the studied temperature range (200–1000 K), indicating that the modulus reduction proceeds at an approximately constant rate, especially at higher temperatures where the exponential term varies slowly. The higher baseline modulus and greater thermal sensitivity in the Y-direction suggest direction-dependent bonding strength and phonon dynamics.

**Fig 14:**
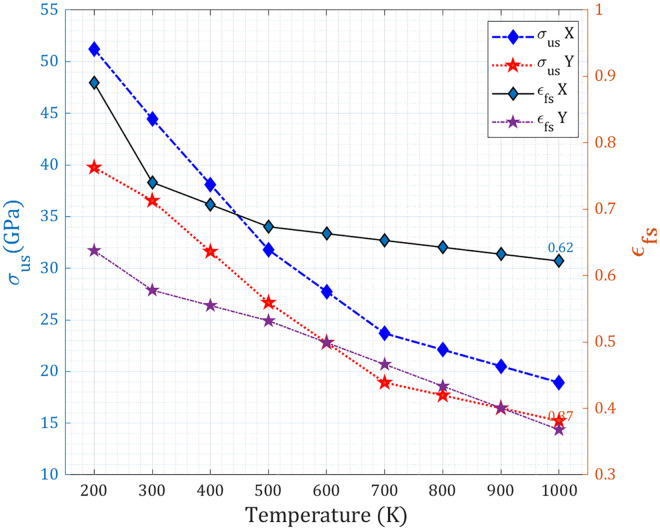
Ultimate stress and fracture strain of a pre-cracked T_4,4,4_-graphyne nanosheet.

The ultimate stress and fracture strain of the pre-cracked T_4,4,4_-graphyne nanosheet exhibit a clear degradation trend with increasing temperature, reflecting the weakening of atomic bonds and enhanced ductility typical of thermally activated fracture behavior. Under uniaxial loading in the X-direction, the ultimate stress decreases from ~51.2 GPa at 200 K to ~18.9 GPa at 1000 K, while the fracture strain reduces moderately from ~0.89 to ~0.62. In the Y-direction, a similar softening occurs, with ultimate stress dropping from ~39.8 GPa to ~15.2 GPa and fracture strain decreasing from ~0.64 to ~0.37 (as shown in Fig 14). The initial higher values in the X-direction confirm a stronger resistance to fracture in that orientation, consistent with more favorable load-bearing paths and crack-bridging mechanisms.

This temperature-dependent degradation is attributed to enhanced thermal vibrations that reduce the cohesive energy between atoms and promote earlier bond breaking under load. At low temperatures, atomic motion is restricted, and the lattice can sustain high stress and deform more extensively before failure. As temperature increases, atoms become more mobile, weakening interatomic forces, promoting crack-tip blunting, and facilitating earlier crack propagation, which lowers both the stress capacity and the strain at break. The more pronounced reduction in fracture strain along the Y-direction highlights its greater thermal sensitivity, likely due to the orientation of weaker or more thermally responsive bonds along that axis. These results confirm the thermally driven brittle-to-quasi-ductile transition in the fracture behavior of anisotropic 2D graphyne nanosheets.

The thermally induced brittle-to-quasi-ductile transition in T_4,4,4_ graphyne nanosheets is quantitatively evidenced by significant changes in fracture strain, ultimate stress, and toughness across the investigated temperature range (200 K to 1000 K). As temperature increases, enhanced thermal vibrations weaken atomic bonds and promote earlier bond breaking, leading to a reduction in both ultimate stress and fracture strain. For instance, in the X-direction, the ultimate stress decreases from approximately 51.2 GPa at 200 K to 18.9 GPa at 1000 K, while the fracture strain moderately reduces from about 0.89 to 0.62. Similarly, in the Y-direction, ultimate stress drops from 39.8 GPa to 15.2 GPa, and fracture strain decreases from 0.64 to 0.37. This reduction in both strength and ductility at higher temperatures is further corroborated by the decline in toughness, representing the material’s energy absorption capacity. In the X-direction, toughness decreases from 12.2 GPa at 200 K to 3.7 GPa at 1000 K, and in the Y-direction, it falls from 10.3 GPa to 2.9 GPa. These quantitative shifts highlight a clear degradation in mechanical performance, consistent with a transition towards a more ductile-like failure mode where the material’s ability to sustain high stress and extensive deformation before failure is diminished. Qualitatively, this transition is also supported by observations of fracture surface morphology, where at lower temperatures or under specific loading conditions (e.g., X-loading), the material exhibits feature such as tortuous crack propagation, ligament bridging, and crack blunting, indicative of more ductile behavior. Conversely, at higher temperatures or under conditions promoting brittle fracture (e.g., Y-loading), crack propagation tends to be more linear with minimal deflection, signifying a more brittle response. These combined quantitative and qualitative analyses provide a comprehensive understanding of the temperature-dependent fracture behavior and the brittle-to-quasi-ductile transition in T_4,4,4_-graphyne nanosheets.

**Fig 15:**
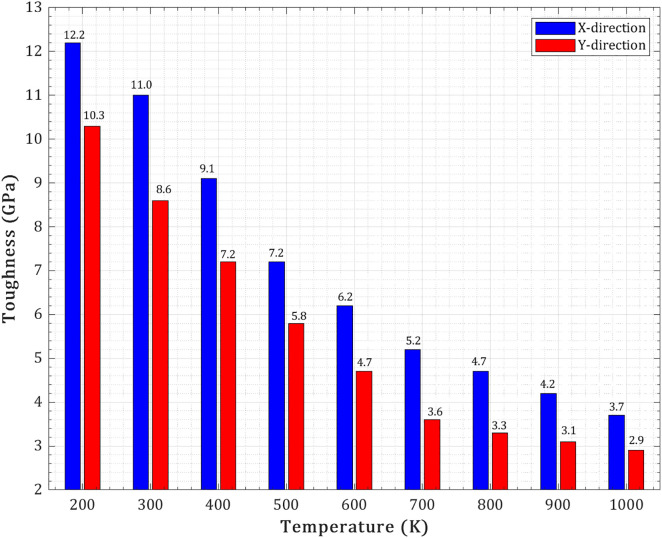
Temperature-dependent variation of toughness for a pre-cracked T_4,4,4_-graphyne nanosheet.

The toughness of the pre-cracked T_4,4,4_-graphyne nanosheet declines consistently with increasing temperature in both loading directions, indicating reduced energy absorption capacity before fracture (as shown in Fig 15). In the X-direction, toughness decreases from ~12.2 GPa at 200 K to ~3.7 GPa at 1000 K, while in the Y-direction it drops from ~10.3 GPa to ~2.9 GPa. This trend reflects the combined influence of decreasing ultimate stress and fracture strain due to thermally induced bond weakening and enhanced atomic mobility. At low temperatures, strong interatomic forces and limited thermal agitation allow the material to store more mechanical energy before failure, contributing to higher toughness. As temperature increases, the ability of the lattice to carry and sustain stress diminishes, leading to lower energy dissipation and earlier crack propagation. The more pronounced toughness in the X-direction across all temperatures underscores the anisotropic fracture resistance of the nanosheet, likely arising from stronger or more interconnected load-bearing bonds in that orientation.

**Fig 16:**
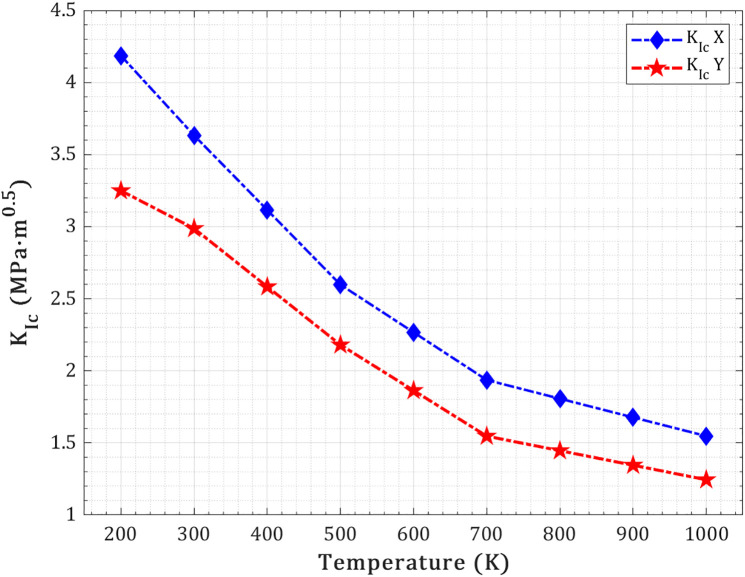
Temperature-dependent variation of mode I fracture toughness for a pre-cracked T_4,4,4_-graphyne nanosheet.

The mode I fracture toughness (${\text{K}}_{\text{Ic}}$) of the pre-cracked T_4,4,4_-graphyne nanosheet decreases significantly with increasing temperature, indicating a reduced resistance to crack initiation and propagation under tensile loading (as shown in Fig 16). For the X-direction, ${\text{K}}_{\text{Ic}}$ drops from ~4.19 MPa.m^0.5^ at 200 K to ~1.55 MPa.m^0.5^ at 1000 K, while in the Y-direction it decreases from ~3.25 to ~1.24 MPa.m^0.5^. This temperature-dependent decline reflects the weakening of atomic cohesion and the loss of lattice stiffness due to increased thermal agitation, which lowers the energy barrier for crack propagation. At lower temperatures, the material’s structure maintains higher rigidity and bond integrity, providing greater resistance to fracture. As temperature rises, bond vibrations increase, atomic mobility enhances, and the crack tip experiences less localized stress shielding, facilitating easier crack growth. The consistently higher ${\text{K}}_{\text{Ic}}$ values in the X-direction suggest that the atomic arrangement along this axis provides more effective crack-bridging and load transfer mechanisms, reinforcing the intrinsic anisotropy in fracture resistance.

## 4. Conclusion

In this study, the mechanical response of pristine and centrally pre-cracked T_4,4,4_-graphyne nanosheets was systematically investigated under uniaxial tension in both principal directions by NEMD based on the AIREBO-M potential. It was found that the elastic moduli decreased nonlinearly with normalized crack length and were well described by a power-law relation, while the angular dependence was accurately modeled using cosine-based Fourier series terms. Ultimate tensile strength and fracture strain were observed to diminish with both increasing crack length and temperature, whereas toughness and mode I fracture toughness exhibited pronounced anisotropy: under X-loading, crack-tip shielding mechanisms such as ligament bridging and bond rotation were activated, enhancing ductility and energy absorption compared to the more brittle Y-direction response. Thermal softening of the elastic modulus was captured by the Wachtman equation and shown to proceed in an approximately linear fashion over 200–1000 K, with a subtle inversion of directional stiffness above 900 K. These findings demonstrated that a critical crack length (~30% of the sheet width) marked the onset of a mechanical percolation threshold, beyond which anisotropic fracture resistance was largely lost and isotropic failure mechanisms predominated. The study therefore established clear relationships between crack geometry, loading orientation, and thermal effects, and highlighted the robust applicability of semi-empirical and trigonometric models for predicting the anisotropic fracture behavior of 2D graphyne sheets.
